# CircRNA and lncRNA-associated competing endogenous RNA networks in medulloblastoma: a scoping review

**DOI:** 10.1186/s12935-024-03427-w

**Published:** 2024-07-15

**Authors:** Fatemeh Nejadi Orang, Mahdi Abdoli Shadbad

**Affiliations:** 1 Immunology Research Center, Tabriz University of Medical Sciences, Tabriz, Iran; 2 Department of Immunology, Faculty of Medicine, Tabriz University of Medical Sciences, Tabriz, Iran

**Keywords:** CeRNA, CircRNA, LncRNA, Medulloblastoma, MiRNA

## Abstract

**Background:**

Medulloblastoma is one of the common primary central nervous system (CNS) malignancies in pediatric patients. The main treatment is surgical resection preceded and/or followed by chemoradiotherapy. However, their serious side effects necessitate a better understanding of medulloblastoma biology to develop novel therapeutic options.

**Main body:**

Circular RNA (circRNA) and long non-coding RNA (lncRNA) regulate gene expression via microRNA (miRNA) pathways. Although growing evidence has highlighted the significance of circRNA and lncRNA-associated competing endogenous RNA (ceRNA) networks in cancers, no study has comprehensively investigated them in medulloblastoma. For this aim, the Web of Science, PubMed, Scopus, and Embase were systematically searched to obtain the relevant papers published before 16 September 2023, adhering to the PRISMA-ScR statement. HOTAIR, NEAT1, linc-NeD125, HHIP-AS1, CRNDE, and TP73-AS1 are the oncogenic lncRNAs, and Nkx2-2as is a tumor-suppressive lncRNA that develop lncRNA-associated ceRNA networks in medulloblastoma. CircSKA3 and circRNA_103128 are upregulated oncogenic circRNAs that develop circRNA-associated ceRNA networks in medulloblastoma.

**Conclusion:**

In summary, this study has provided an overview of the existing evidence on circRNA and lncRNA-associated ceRNA networks and their impact on miRNA and mRNA expression involved in various signaling pathways of medulloblastoma. Suppressing the oncogenic ceRNA networks and augmenting tumor-suppressive ceRNA networks can provide ample opportunities for medulloblastoma treatment.

**Supplementary Information:**

The online version contains supplementary material available at 10.1186/s12935-024-03427-w.

## Introduction

Medulloblastoma is recognized as the most prevalent form of brain tumor among children, accounting for nearly 20% of all brain tumors found in pediatric patients [1]. Medulloblastoma is most commonly diagnosed before age 15 and has two incidence peaks between the ages of 3–4 and 8–9 [2]. The occurrence of this tumor in older patients is rare, comprising less than 1% of all primary CNS tumors in adults [3]. According to the latest classification scheme, medulloblastoma is divided into two distinct categories, i.e., histologically defined and genetically defined. Histologically, medulloblastoma can be categorized into different types, including classic, desmoplastic/nodular (DN), and large cell/anaplastic (LCA). Genetic classification divides it into four molecular subtypes, i.e., wingless (WNT), sonic hedgehog (SHH), group 3, and group 4. Each of these subtypes has distinct clinical and molecular characteristics [4]. The primary therapeutic strategies for medulloblastoma include a combination of surgical resection, radiotherapy, and chemotherapy. However, clinical outcomes have been not desirable, and 5-year survival rates range between 60 and 80% [5, 6]. Hence, an in-depth investigation of the molecular mechanisms involved in the pathogenesis of medulloblastoma is essential to improve patients' prognoses and clinical outcomes.

Non-coding RNAs (ncRNAs) are a class of RNAs that lack the ability to encode functional proteins [7]. Since their discovery, the biological significance of ncRNAs has become increasingly evident, which has led to a shift in the perspective of RNA from being a simple intermediary of protein synthesis to being a functional molecule with essential roles in regulating gene expression and genome organization [8]. In recent years, studies have indicated that these ncRNAs play vital regulatory roles in the initiation and progression of various types of cancer [7]. In line with this, it has been demonstrated that the expression levels of various ncRNAs are notably different between medulloblastoma and normal cerebellar cells [9]. NcRNAs can be classified into various classes based on size and function. The three primary classes of regulatory ncRNAs are microRNAs (miRNAs), long non-coding RNAs (lncRNAs), and circular RNAs (circRNAs). Owing to their intrinsic characteristics, they may exhibit tissue or disease specificity and can be detected in all bodily fluids, which makes them potentially desirable to be used as biomarkers [10]. LncRNAs are a class of RNA molecules that are longer than 200 nucleotides in length [11]. Through multiple mechanisms, they play a crucial role in regulating gene expression at various levels, including epigenetic, transcriptional, and post-transcriptional regulation. This regulatory role holds particular significance in the central nervous system (CNS) [12]. Most lncRNAs are presumed to be transcribed and processed similarly to mRNAs. They are primarily transcribed by RNA polymerase II and often possess 5′-end m7G caps and 3′-end poly(A) tails [13]. Regarding the chromosomal position, lncRNAs are classified into promoter-associated lncRNAs, antisense, intronic, enhancer RNAs, divergent, intergenic, and transcription start site-associated lncRNAs [14]. LncRNAs function as competing endogenous RNAs (ceRNAs) within a regulatory network by serving as a "sponge" for target miRNAs [15]. Circular RNAs (circRNAs) are single-stranded RNA transcripts with a covalently closed circular structure. They are produced through an alternative splicing process and lack the 5′ caps and 3′ poly(A) tails; this structural feature in circRNAs renders them resistant to degradation by ribonucleases [16]. Furthermore, most circRNAs exhibit evolutionary conservation across various species [17]. CircRNAs are generated through the transcription of precursor mRNA (pre-mRNA) by RNA polymerase II. They can function as molecular sponges for miRNAs, thus regulating their biological activities [18]. Recent evidence has indicated that aberrant expression of circRNAs occurs in various types of cancer, including breast cancer [19], pancreatic ductal adenocarcinoma [20], bladder carcinoma [21], glioblastoma [22], and medulloblastoma [23]. MiRNAs are a class of short, single-stranded RNA molecules comprising 18–22 nucleotides that exert a significant regulatory role in gene expression at the post-transcriptional level [24]. In the last decade, accumulating studies have been dedicated to the assessment of miRNA expression, revealing significant alterations in their expression profiles in different diseases [25–28]. A single miRNA can have multiple direct targets; therefore, a dysregulated miRNA expression can dysregulate a wide range of crucial signaling pathways [29]. The conventional miRNA biogenesis pathway follows a two-step process involving nuclear and cytoplasmic cleavage events. Nonetheless, there are alternative biogenesis pathways that vary in the number of cleavage events and the responsible enzymes [30]. MiRNAs are initially transcribed by RNA polymerase II in the nucleus, leading to the formation of primary miRNA (pri-miRNAs) transcripts with stem-loop structures [31]. Subsequently, the microprocessor complex, comprising the RNase III enzyme Drosha and its cofactor DGCR8, cleaves pri-miRNA to generate a precursor miRNA (pre-miRNA) [32]. Interacting with RanGTP/Exportin-5 transports pre-miRNA from the nucleus to the cytoplasm [33]. In the cytoplasm, the RNase III enzyme Dicer recognizes pre-miRNAs and cleaves them into mature duplex miRNA, which later unwinds into two separate strands, the guide strand and the passenger strand [29, 34]. The guide strand is integrated into the RNA-induced silencing complex (RISC) and guides RISC to complementary target mRNAs for post‑transcriptional gene silencing [29].

CeRNA networks are composed of a group of RNA molecules that play a significant role in gene regulation. These ceRNAs share miRNA recognition elements (MREs), thereby regulating each other [35]. In this regard, circRNA and lncRNA-mediated ceRNA have been extensively studied in various cancers [36–38]. However, there is no comprehensive study that systematically reviews the current knowledge on the significance of these networks in medulloblastoma. This scoping review presents current evidence on the identified circRNA and lncRNA-mediated ceRNA networks and their significance in medulloblastoma development. These insights can pave the way for developing novel therapeutic and biomarker tools for affected patients.

## Method

### Scoping review protocol

The guidelines for preferred reporting items for systematic reviews and meta-analyses extension for scoping reviews (PRISMA-ScR) are followed by the present scoping review [39] (Supplementary data). The five steps of the present scoping review include formulating the research question, identifying the relevant publications, selecting studies, charting the data, and summarizing and disclosing the findings.

### Research question

Given the significant role of the ceRNA networks in the regulation of gene expression, the present study aimed to comprehensively review the current knowledge on the circRNA and lncRNA-associated ceRNA networks in medulloblastoma development.

### Relevant publication identification

The Web of Science, PubMed, Scopus, and Embase were systematically searched to find the relevant studies published before 16 September 2023; the systematic searches did not have any restriction on language, country, or time. LncRNA, circRNA, miRNA, ceRNA, medulloblastoma, and their different versions, along with the Emtree and medical subject headings (MeSh) terms, were used for the systematic search.

### Study selection

After retrieving the publications from the above-mentioned databases and removing duplicated records, the papers were reviewed in two phases. In the first phase, the title and abstract of the obtained studies were reviewed. In the second phase, the full texts of the remaining papers were thoroughly reviewed. The criteria for inclusion were the following. First, the included study must be an original paper published in English. Second, the included study must study the interaction between lncRNA with miRNA or circRNA with miRNAs in medulloblastoma. Third, the experimental study must contain at least one of the human medulloblastoma cell lines.

### Data charting

The studied ncRNAs and the related axis, medulloblastoma cell line, and the effect of the studied axis on medulloblastoma formation were all extracted from the included studies.

### Summarizing and reporting the obtained results

The present scoping review summarizes the results of the studies that were included and also investigates the effect of the identified miRNAs, circRNAs, and lncRNAs on the development of medulloblastoma that were not present in the included studies.

### In silico study

To extend the understanding of the impact of ceRNA on cellular singling pathways, miRPathDB v2.0 was used to access the Reactome database. A minimum of two significant miRNAs per pathway and strong experimental evidence were the criteria for the related analysis.

## Results

### Systematic search results

**Fig. 1:**
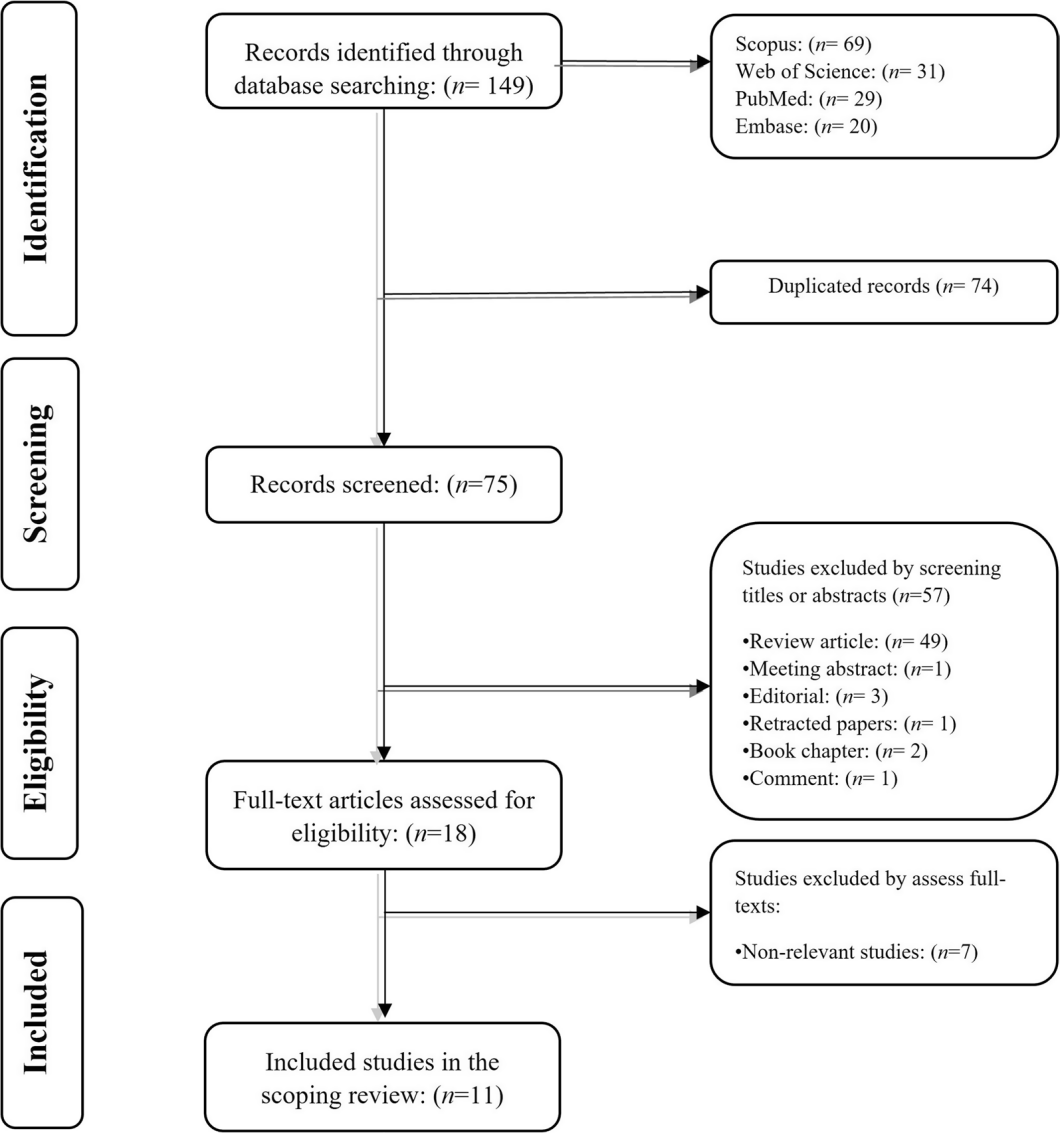
The flowchart of the study

The systematic search on Web of Science, PubMed, Scopus, and Embase identified 149 papers published before 16 September 2023. After removing duplicated studies, 57 papers were also excluded based on reviewing their title and abstracts. Ultimately, seven papers were excluded from the present scoping review because they did not meet the above-mentioned inclusion criteria in the full-text assessment phase. Figure 1 shows the study's flowchart.

### The characteristics of the included papers

The included studies were published between 2017 and 2023. Daoy was the most studied medulloblastoma human cell line. According to the ATCC, this cell line was obtained from a 4-year-old white male with desmoplastic cerebellar type. HOTAIR, NEAT1, linc-NeD125, HHIP-AS1, CRNDE, and TP73-AS1 are the identified oncogenic lncRNA in medulloblastoma, and Nkx2-2as is a tumor-suppressive lncRNA in medulloblastoma. Based on the current experimental evidence, circSKA3, which sponges miR-326, miR-520 h, and miR-383-5p, and circRNA_103128, which sponges miR-129-5p, are upregulated oncogenic circRNA in medulloblastoma (Table 1).

**Table 1:** The characteristics of the included studies

No	Identified axis	Cell line	Effect on medulloblastoma	References
1	HOTAIR/miR-1-3p and miR-206/ YY1	Daoy and D283	Stimulated oncogenic lncRNA in medulloblastoma	Zhang et al. [42]
2	NEAT1/miR-23a-3p/GLS	Daoy and D341	Stimulated oncogenic lncRNA in medulloblastoma	Ge et al. [44]
3	Linc-NeD125/miR-19a-3p, miR-19b-3p, miR-106a-5p/CDK6, MYCN, SNCAIP, and KDM6A	D283 and CHLA-01	Stimulated oncogenic lncRNA in group 4 medulloblastomas	Laneve et al. [52]
4	HHIP-AS1/miR-425-5p/ DYNC1I2	Daoy	Oncogenic lncRNA in medulloblastoma	Bartl et al. [55]
5	CRNDE/miR-29c-3p	Daoy and D341	Stimulated oncogenic lncRNA in cisplatin-treated medulloblastoma	Sun et al. [57]
6	TP73-AS1/miR-494-3p/ ELF5A2	Daoy and D341	Stimulated oncogenic lncRNA in medulloblastoma	Li et al. [60]
7	Nkx2-2as/miR-103a-3p, miR-107 and miR-548 m /BTG2, LATS1, and LAST2	Daoy, D341, and HEK293T	Tumor-suppressive lncRNA in medulloblastoma	Zhang et al. [61]
8	CircSKA3/miR-326/ID3	Daoy and D283	Stimulated oncogenic circRNA in medulloblastoma	Zhao et al. [64]
9	CircSKA3/miR-520 h/ CDK6	Daoy	Stimulated oncogenic circRNA in medulloblastoma	Liu et al. [65]
10	CircSKA3/miR-383-5p/ FOXM1	Daoy and ONS-76	Stimulated oncogenic circRNA in medulloblastoma	Wang et al. [63]
11	CircRNA-103128/miR-129-5p/SOX4	Daoy	Stimulated oncogenic circRNA in medulloblastoma	Yin et al. [66]

*HOTAIR* Hox transcript antisense intergenic RNA, *NEAT1* Nuclear paraspeckle assembly transcript 1, *CDK6* Cyclin-dependent kinase 6, *SNCAIP* synuclein alpha interacting protein, *KDM6A* Lysine demethylase 6A, *HHIP-AS1* HedgeHog Interacting Protein-AntiSense 1, *DYNC1I2* Dynein cytoplasmic 1 intermediate chain 2, *CRNDE* Colorectal neoplasia differentially expressed, *TP73-AS1* Tumour protein P73 antisense RNA 1, *ELF5A2* Eukaryotic Initiation Factor 5A, *BTG2* B-cell translocation gene 2, *LATS1* large tumor suppressor kinase 1, *LAST2* large tumor suppressor kinase 2, *circSKA3* circRNA spindle and kinetochore associated complex subunit 3, *ID3* inhibitor of DNA binding 3, *FOXM1* Forkhead box M1, *SOX4* SRY-box transcription factor 4

### The enrichment analysis

**Fig. 2:**
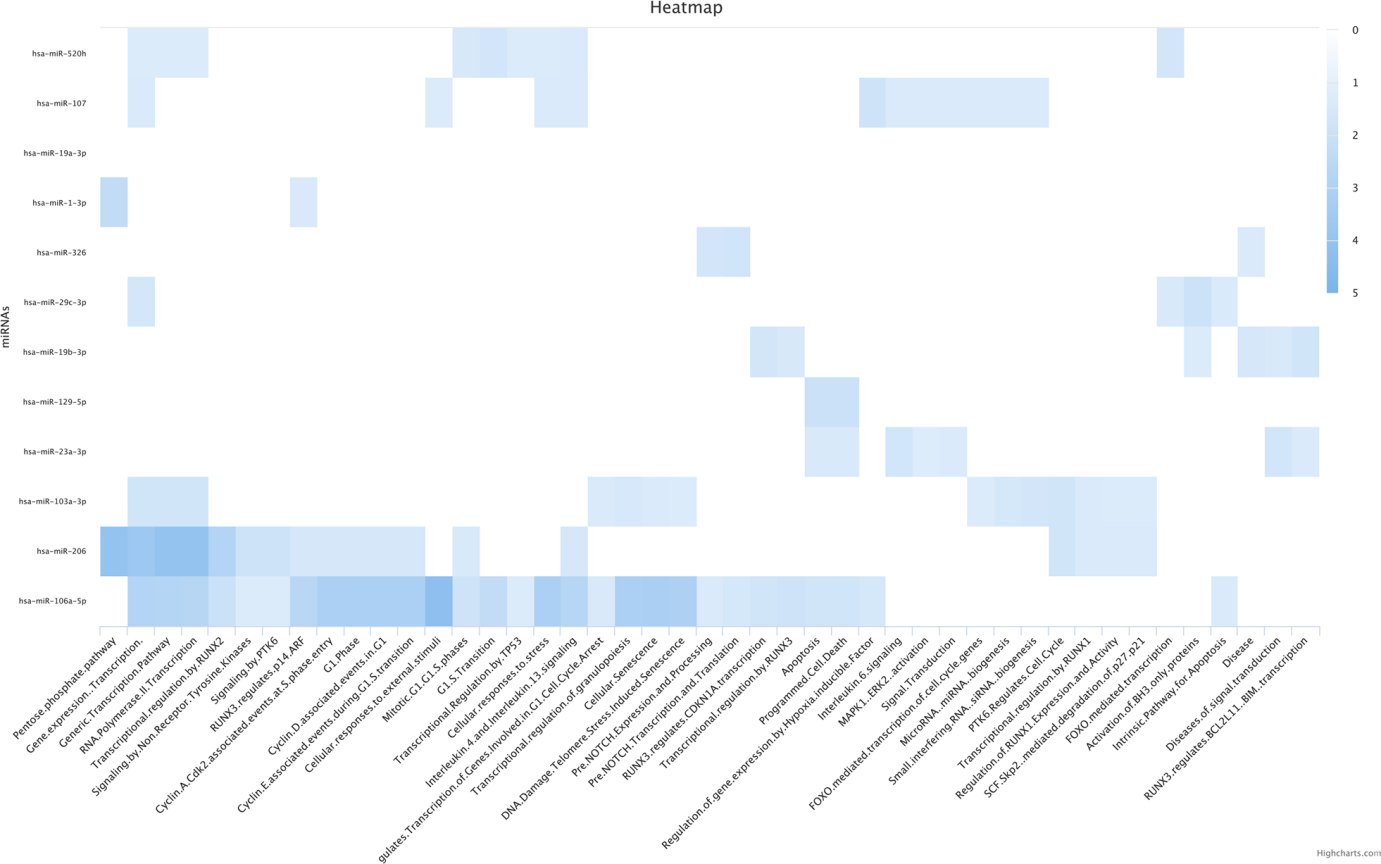
The enrichment analysis of studied microRNAs

Based on the Reactome database, the identified miRNAs regulate various cellular pathways, like cell cycle, apoptosis, MAPK1/ERK2 pathway, etc. For instance, miR-106a-5p, miR-23a-3p, and miR-129-5p are enriched for apoptosis (Fig. 2).

## Discussion

Despite the recent advances in our understanding of medulloblastoma biology, the clinical outcome of affected patients is still unfavorable. A better understanding of ncRNAs and ceRNAs might provide valuable insights regarding treating medulloblastoma [40]. The following discusses the current evidence on the significance of circRNA and lncRNA-associated ceRNA networks in medulloblastoma.

### HOTAIR-mediated ceRNA

As a located lncRNA on chromosome 12, homeobox transcript antisense intergenic RNA (HOTAIR) can interact with PRC2, LSD1, and miRNAs, leading to gene expression regulation [41]. Zhang et al. have reported that HOTAIR expression level is substantially upregulated in medulloblastoma tissues and cell lines compared with non-tumoral ones. HOTAIR knockdown improves apoptosis rate, decreases the cell viability, clonogenicity, migration, and invasion, and reduces tumor volume in animal models; this oncogenic effect is medicated via the HOTAIR/miR-1-3p and miR-206/ YY1 axes in medulloblastoma. Also, the ectopic expression of miR-1-3p and miR-206 has been associated with decreased tumor growth in animal models [42]. In line with this, it has been shown that miR-206 is downregulated in medulloblastoma tissues, and its increased expression decreased the cell viability and migration of medulloblastoma cells via the miR-206/LASP1 axis [43].

### NEAT1-mediated ceRNA

As a component of nuclear paraspeckles, nuclear-enriched abundant transcript 1 (NEAT1) is located on chromosome 11q13.1; this lncRNA is dysregulated in various cancers, like glioma and medulloblastoma [44, 45]. Ge et al. have shown that NEAT1 knockdown increases the chemosensivity of medulloblastoma cells and potentiates cisplatin-mediated apoptosis activation. This chemoresistance of medulloblastoma cells is mediated via the NEAT1/miR-23a-3p/GLS axis [44]. In line with this, the *in-silico* results have shown that miR-23a-3p is enriched for apoptosis. Apoptosis is a type of regulated cell death that has two pathways, i.e., intrinsic and extrinsic pathways [46]. In the intrinsic pathway, the insertion of BAX and BAK into the mitochondrial membrane releases cytochrome c, leading to the activation of caspase-9 and executioner caspases. In the extrinsic pathway, the death-inducing signaling complex results in the activation of caspase-8, caspase-10, caspases-3, caspases-6, and caspases-7; these events stimulate apoptosis activation [47, 48]. In addition, the *in-silico* results have shown that miR-23a-3p is enriched for the MAPK pathway as well. The MAPK/ERK pathway is among the activated oncogenic pathways in medulloblastoma and its blockade is associated with decreased proliferation, stemness, and invasion in medulloblastoma cells [49, 50]. Also, it has been reported that NEAT1 knockdown increases the chemosensitivity of glioblastoma cells to temozolomide [51].

### Linc-NeD125-mediated ceRNA

Linc-NeD125 is a long intergenic ncRNA that is located on chromosome 11. Laneve et al. have reported that linc-NeD125 expression level is substantially increased in G4 medulloblastoma, and its knockdown decreases the proliferation of G4 medulloblastoma cells and downregulates the protein expression of CDK6, MYCN, SNCAIP, and KDM6A via the linc-NeD125/miR-19a-3p, miR-19b-3p, miR-106a-5p/CDK6, MYCN, SNCAIP, and KDM6A axes [52]. Consistent with this, the *in-silico* results have shown that miR-106a-5p is enriched for the G1 phase, cyclin D-associated events at the G1 phase, cyclin E-associated events during G1-S transition, cyclin A-CDK2 associated events at S phase entry as well as apoptosis. The complex of cyclin D with CDK4/CDK6 causes the RB-phosphorization and RB-phosphorization-mediated E2F release, leading to the transition from the G1 phase to the S phase. Cyclin E1 and E2 bind and activate CDK2, leading to RB and p27^KIP1^ phosphorylation. The cyclin E/CDK2 active complex leads to the S phase initiation and cyclin A/CDK2 is formed near the end of the S phase and leads to cell cycle progression from the S phase to the G2 phase. The cyclin A/CDK1 complex results in M phase entry [53]. However, it has been reported that the ectopic expression of linc-NeD125 suppresses the proliferation of neuroblastoma cells as well [54].

### HHIP-AS1-mediated ceRNA

Hedgehog interacting protein-antisense 1 (HHIP-AS1) is a lncRNA located on chromosome 4. Bartl et al. have shown that HHIP-AS1 knockdown decreases the cell viability and proliferation of tumoral cells and increases the survival of medulloblastoma models by altering the mitotic spindle organization; the proliferative effect of HHIP-AS1 is mediated through the HHIP-AS1/miR-425-5p/DYNC1I2 axis [55].

### CRNDE-mediated ceRNA

Colorectal neoplasia differentially expressed (CRNDE) is a lncRNA located on chromosome 16. It has been reported that CRNDE expression level is elevated in medulloblastoma tissues compared to adjacent non-tumoral tissues, and its knockdown arrests the cycle at the S phase, activates apoptosis rate, inhibits clonogenicity, reduces the proliferation of medulloblastoma cells in vitro. CRNDE knockdown also decreases tumor growth in animal models of medulloblastoma [56]. Consistent with this, Sun et al. have shown that CRNDE knockdown or miR-29c-3p ectopic expression decreases migration, invasion, clonogenicity, and proliferation and increases the apoptosis of medulloblastoma cells and inhibits tumor growth in animal models of medulloblastoma via the CRNDE/miR-29c-3p axis. Besides, the increased expression of miR-29c-3p has been associated with improved chemosensitivity of medulloblastoma cells to cisplatin [57].

### TP73-AS1-mediated ceRNA

LncRNA TP73-AS1 is located on chromosome 1, and its expression is dysregulated in cancers like medulloblastoma and lung adenocarcinoma [58, 59]. Li et al. have shown that TP73-AS1 expression level is increased in medulloblastoma tissues compared to non-tumoral tissues, and TP73-AS1 knockdown decreases the proliferation, migration, and invasion and enhances the apoptosis of medulloblastoma cells via the TP73-AS1/miR-494-3p/ELF5A2 axis. TP73-AS1 knockdown also reduces tumor growth in animal models of medulloblastoma [60]. Increased expression of TP73-AS1 has been associated with poor prognosis of TP53 wild-type SHH medulloblastoma patients, and its knockdown increases the apoptosis rate, decreases migration, reduces proliferation, and increases survival of animal models [59].

### Nkx2-2as-mediated ceRNA

Nkx2-2as is a lncRNA that is located on chromosome 20. Zhang et al. have shown that Nkx2-2as decreases the proliferation, clonogenicity, invasion, and tumor sphere of medulloblastoma cells via the Nkx2-2as/miR-103a-3p, miR-107, and miR-548 m/BTG2, LATS1 and LAST2. In animal models of medulloblastoma, Nkx2-2as ectopic expression decreases the tumor growth, and the administrating intracerebellar of Nkx2-2as lentiviruses increases the survival of affected mice [61]. Besides, the *in-silico* results have shown that miR-107 is enriched for the MAPK/ERK pathway.

### circSKA3-mediated ceRNA

CircRNA spindle and kinetochore-associated complex subunit 3 (circSKA3) is dysregulated in cancers like breast cancer and medulloblastoma [62, 63]. Wang et al. have reported that circSKA3 is considerably upregulated in medulloblastoma tissues, and circSKA3 silencing or miR-383-5p ectopic expression decreases the cell viability, arrests the cell cycle at sub-G1 phase, enhances the apoptosis, and reduces the migration and invasion of medulloblastoma cells via the circSKA3/miR-383-5p/FOXM1 axis. In addition, in vivo results have demonstrated that circSKA3 silencing decreases tumor weight in animal models of medulloblastoma [63]. Zhao et al. have reported comparable results regarding the oncogenic nature of circSKA3 leveraging both in vitro and in vivo assays and highlighted the circSKA3/miR-326/ID3 axis in medulloblastoma [64]. In line with these, Liu et al. have demonstrated that circSKA3 overexpression enhances cell viability, increases the migration and invasion of medulloblastoma cells, and results in cell cycle progression via the circSKA3/miR-520 h/CDK6 axis [65].

### circRNA-103128-mediated ceRNA

circRNA_103128, also known as has_circ_0061694, is located on chromosome 21, and its expression level is increased in medulloblastoma tissues; Yin et al. have reported that circRNA-103128 knockdown is associated with increased apoptosis rate, reduced cell viability, migration, invasion, and clonogenicity of medulloblastoma cells, and decreased tumor weight in animal models via the circRNA_103128/miR-129-5p/SOX4 [66]. The *in-silico* results have shown that miR-129-5p is enriched for apoptosis. Also, miR-129-5p mimics have anti-tumoral effects in terms of decreasing cell viability and arresting the cell cycle in glioblastoma cells as well [67].

## Expert and topical summary

The circRNA and lncRNA-associated ceRNA topic is an emerging topic in medulloblastoma. For this reason, the present scoping review was conducted to study the extent and scope of research conducted on this topic. Given the fact that this topic is relatively new, the extent of research on medulloblastoma is relatively smaller than on glioma; therefore, further studies are needed to facilitate the application of ceRNA-related therapy for medulloblastoma.

The current study has several strengths. First, it investigated the scope of circRNA and lncRNA-associated ceRNA networks in medulloblastoma development and highlighted their significance. Second, this study benefited from *in-silico* studies on the identified miRNAs as well. However, this study suffers from a limitation as well. The protocol of this study was not registered. Overall, the current evidence indicates that HOTAIR, NEAT1, linc-NeD125, HHIP-AS1, CRNDE, and TP73-AS1 are oncogenic lncRNAs and Nkx2-2as is a tumor-suppressive lncRNA that forms lncRNA-associated ceRNAs in medulloblastoma. Also, circSKA3 and circRNA-103128 are oncogenic circRNAs that have circRNA-mediated ceRNA in medulloblastoma. Targeting oncogenic ceRNAs and ectopic expression of tumor-suppressive ones can be a promising approach for treating medulloblastoma.

### Supplementary Information

Supplementary material 1.

## Data Availability

The datasets analyzed during the current study for enrichment analyses are available in the miRPathDB V 2.0 (https://mpd.bioinf.uni-sb.de/overview.html).

## Abbreviations

CNS: Central nervous system
circRNA: Circular RNA
lncRNA: Long non-coding RNA
miRNA: MicroRNA
ceRNA: Competing endogenous RNA
DN: Desmoplastic/nodular
LCA: Large cell/anaplastic
WNT: Wingless
SHH: Sonic hedgehog
pri-miRNAs: Primary miRNA
RISC: RNA-induced silencing complex
MREs: MiRNA recognition elements
PRISMA-ScR: Preferred reporting items for systematic reviews and meta-analyses extension for scoping reviews
HOTAIR: Homeobox transcript antisense intergenic RNA
HHIP-AS1: Hedgehog interacting protein-antisense 1
CRNDE: Colorectal neoplasia differentially expressed
MAPK: Mitogen-activated protein kinase
ERK: Extracellular signal-regulated kinase
